# Piezoelectric Sensing Techniques in Structural Health Monitoring: A State-of-the-Art Review

**DOI:** 10.3390/s20133730

**Published:** 2020-07-03

**Authors:** Pengcheng Jiao, King-James I. Egbe, Yiwei Xie, Ali Matin Nazar, Amir H. Alavi

**Affiliations:** 1 Institute of Port, Coastal and Offshore Engineering, Ocean College, Zhejiang University, Zhoushan 316021, China; pjiao@zju.edu.cn (P.J.); ekjames@zju.edu.cn (K.-J.I.E.); ali.matinnazar@zju.edu.cn (A.M.N.); 2 Engineering Research Center of Oceanic Sensing Technology and Equipment, Zhejiang University, Zhoushan 316021, China; 3 Centre for Optical and Electromagnetic Research, State Key Laboratory for Modern OpticalInstrumentation, Zhejiang Provincial Key Laboratory for Sensing Technologies, Zhejiang University, Hangzhou 310058, China; yiweixie@zju.edu.cn; 4 Department of Civil and Environmental Engineering, University of Pittsburgh, Pittsburgh, PA 15261, USA; 5 Department of Computer Science and Information Engineering, Asia University, Taichung, Taiwan

**Keywords:** smart civil infrastructure, structural health monitoring, piezoelectric, electromechanical impedance, ultrasonic lamb wave, energy harvesting

## Abstract

Recently, there has been a growing interest in deploying smart materials as sensing components of structural health monitoring systems. In this arena, piezoelectric materials offer great promise for researchers to rapidly expand their many potential applications. The main goal of this study is to review the state-of-the-art piezoelectric-based sensing techniques that are currently used in the structural health monitoring area. These techniques range from piezoelectric electromechanical impedance and ultrasonic Lamb wave methods to a class of cutting-edge self-powered sensing systems. We present the principle of the piezoelectric effect and the underlying mechanisms used by the piezoelectric sensing methods to detect the structural response. Furthermore, the pros and cons of the current methodologies are discussed. In the end, we envision a role of the piezoelectric-based techniques in developing the next-generation self-monitoring and self-powering health monitoring systems.

## 1. Introduction

Structural health monitoring (SHM) systems are vital elements of smart civil infrastructure. At its core, SHM is a systematic identification of damage in a structure using time-varying data outputted over time [1,2]. The SHM systems make use of sensing technologies to track and evaluate the symptoms of operational incidents, anomalies, and deterioration or damage indicators that may affect the operation, serviceability, safety or reliability [3,4]. There has been a growing trend towards developing robust SHM methods for continuous monitoring, acquisition, validation and analysis of technical data to facilitate life-cycle management decisions [2,5,6,7]. A number of SHM methods based on different principles were developed in the last three decades [8,9,10]. These methods have been used for both global and local damage detection. Global damage detection deals with the overall structural response, e.g., changes in mode shape and natural frequencies of the structure. Local damage detection is focused on screening structures at small scales of components and sub-components for damages (e.g., cracks, rust) [7]. Wireless sensor networks have revolutionized the traditional high-cost wired SHM systems due to their ease of placement, high spatial resolution and wireless data transmission [11,12,13]. Despite their efficiency, the wireless SHM systems still have some limitations, such as difficulties implementing large-scale sensor networks, accurate damage localization, and a lack of appropriate principles to manage and interpret the data from extensive sensor networks [14]. The other field that has witnessed significant growth within the SHM area is deploying smart materials as sensing components. Smart materials work with fundamentally different principles from conventional materials and exhibit greater sensitivity to any changes in their surrounding environment. Smart materials possess adaptive capabilities to external stimuli, such as loads or the environment, with inherent intelligence. Smart materials include optical fibers, piezoelectric polymers and ceramics, electro-rheological (ER) fluids, magneto-strictive materials and shape memory alloys (SMAs). Among the various smart material types, piezoelectric materials have received notable attention for the SHM of civil infrastructure systems due to their special ability to respond to stimuli, embeddability and compatibility with construction materials [15,16,17,18,19,20,21]. Piezoelectric materials are cost-efficient and can sense ambient vibrations in the host structures. By tuning the piezoelectric mode of operation, researchers have applied it in the design of advanced sensors such as a piezoelectric-excited guided wave and piezoelectric electromechanical impedance sensors [16]. Furthermore, recent studies in the area of self-powered piezoelectric-floating gate technology have shown the potential of using piezoelectric materials in securing a sustainable powering source for sensor networks [15].

In this paper, we review the recent advances of piezoelectric sensing technologies for SHM. We classify the piezoelectric sensors according to their sensing and design functionalities. Further discussion is presented about the laboratory and field testing of these sensors. In the end, we envision the piezoelectric-based technique as an intrinsic part of next-generation SHM, which can self-monitor structural systems and predict the occurrence of failures in advance. The rest of the paper has been organized as follows: Section 2 introduces the principle of piezoelectricity and the basic coupling equations. Section 3 discusses the piezoelectric impedance method using practical research examples. Section 4 explains the Lamb-wave-based process for piezoelectric-based SHM. Section 5 introduces a fully self-powered piezo-floating gate technology. Section 6 and Section 7 present the future trends and concluding remarks, respectively.

## 2. Principle of Piezoelectric Sensing in SHM

In the last two decades, there has been growing interest in developing new sensing technologies for SHM. The widely used piezoelectric methods use the piezoelectric principles for passive and active monitoring of the structural systems [22]. The piezoelectric effect is a mutual coupling between electrical and mechanical variables in the material (mechanical stress, strain, and electrical field or charge). This effect can be described by the following linear constitutive equations [23]

(1) $$S_{\mathrm{ij}}=S^{E}{}_{\mathrm{ijkl}}T_{\mathrm{kl}}+d_{\mathrm{ijm}}E_{m}$$

and

(2) $$D_{n}=d_{\mathrm{nkl}}T_{\mathrm{kl}}+\varepsilon^{T}{}_{\mathrm{mn}}E_{m}$$

where *S*_ij_, *T*_kl,_ *D*_n,_ and *E*_m_ correspond to the mechanical strain, stress tensors, electric displacement, and field vectors, respectively. Although the constitutive equations of piezoelectricity are in tensor form, a piezoelectric device can often be described by scalar equations. Under the quasi-static assumption, which neglects the dynamic behavior at the resonance of the device, linear, frequency-independent equations that represent the device are written as follows [23,24,25]

(3) $$\partial =S^{V}F+dV$$

and

(4) $$Q=dF+C^{F}V$$

where,

∂: Deflection or displacement of the piezoelectric device;
Q: Charge on the terminals of the piezoelectric device;
F: Force exerted on the device;
V: Voltage across the electrodes;
SV: Compliance under constant voltage;
d: Piezoelectric coefficient;
CF: Capacitance under constant force.

**Figure 1:**
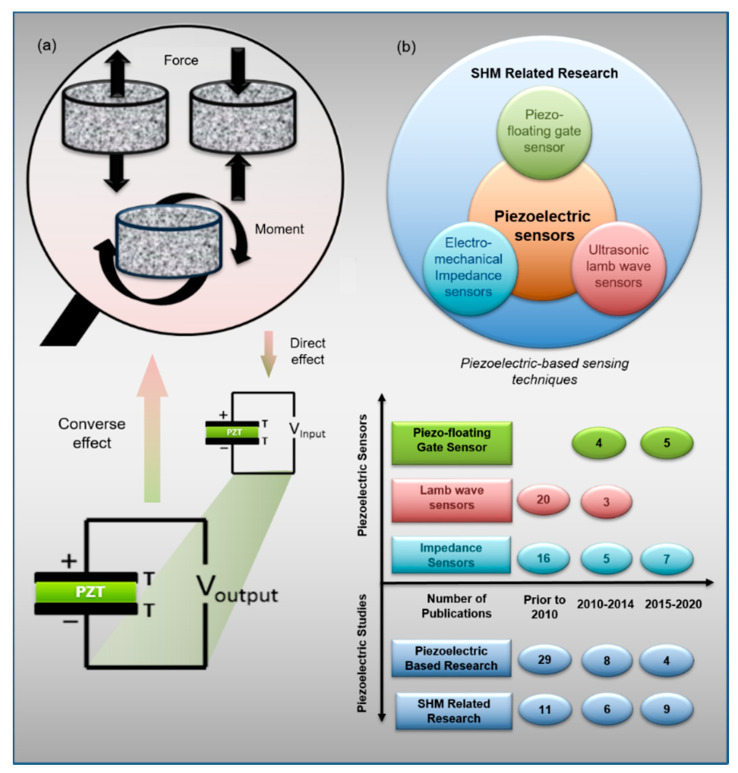
(**a**) Principle of the direct and inverse response in the piezoelectric effect, and (**b**) Summary of the studies in the piezoelectric-enabled sensing techniques in structural health monitoring (SHM).

In general, piezoelectricity implies the production of an electrical charge from a piezoelectric material when stressed mechanically [26,27]. A reverse mechanism is also true, as a mechanical strain/stress is produced when an electrical field/charge is applied. Figure 1a demonstrates the piezoelectric sensing process, utilizing the direct or inverse response of the piezoelectric effect to monitor structures [24,28,29]. In this context, piezoelectric materials have been used in different sensing devices as actuators, sensors, or both [24,27]. Figure 1b provides an overview of some piezoelectric-enabled sensing technologies in SHM and the reviewed studies. Table 1 summarizes the existing studies regarding the sensor type, mode of sensing, and principle of operation of the piezoelectric sensing techniques in SHM.

## 3. Piezoelectric Impedance Sensing Technique

**Figure 2:**
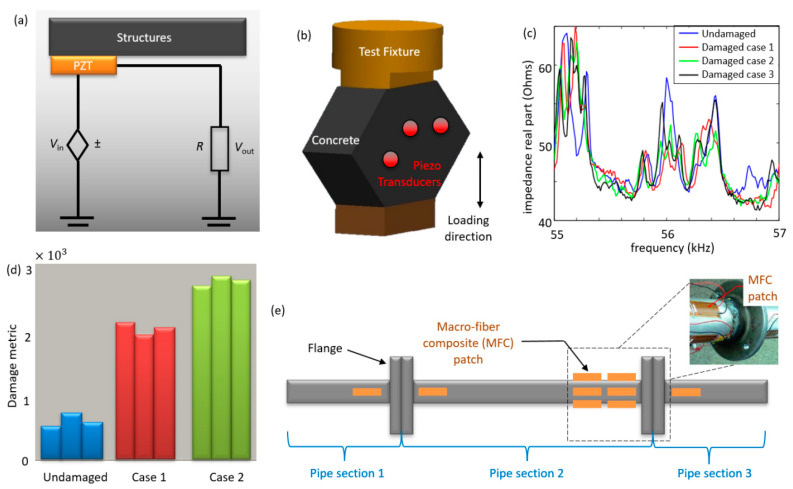
(**a**) Sketch of the Lead Zircontate Titanate (PZT) circuit showing the relation of PZT electrical impedance [38], (**b**) Loading configuration and placement of the PZTs [38], (**c**) Impedance (real part) versus frequency chart [38,39], (**d**) Damage metric for PZT transducers. The damage metric value increases if the damage is located close to the sensor [38], and (**e**) Pipe section showing the locations of the MFC patch [40].

This section reviews the sensing techniques based on the impedance measurement of the host structure using piezoelectric transducers such as Lead Zirconate Titanate (PZT), and macro-fiber composite (MFC) [24,25,26,27,30,31,32,33]. PZT is a ceramic piezoelectric material capable of detecting changes in pressure, acceleration, temperature, strain, or force by converting these energy forms into an electrical charge or signal and vice versa [24,31,35]. Impedance measurement application for the monitoring of structural health has developed significantly and has been applied in different forms [34,35], as shown in Figure 2.

### 3.1. Design Principle and Paradigms

Electromechanical impedance or impedance measurements are based on variations in a structure’s resistance to deformation (elastic modulus) [75]. This method comprises two phases: actuation and sensing. When a piezoelectric transducer is triggered harmonically (10–500 Hz) by an electric field (1–10 V) (actuation phase), it imparts a proportional harmonic force to its host structure. The reflections by the host structure back to the PZT in response to this disturbance (sensing phase) create a signature called electromechanical “impedance” or the inverse “admittance”, depending on the parameter under consideration. The degree of reflection is dependent on the stiffness or elastic modulus of the structure, assuming that the same impact force is applied with each measurement [24]. A baseline measurement of the healthy structure can be carried out and compared with another measurement at another time in the future to check for damage. The possibility of variations in impedance measurements that do not reflect damage exist [75]. These can be triggered by the bonding conditions with structural and external vibrations [27].

The underlying principle behind this sensing technique is the energy exchange between the sensor and the monitored structure, as shown in Figure 2a [31,34,75]. The electrical impedance of the sensor is proven to be directly related to the mechanical impedance of the structural unit where the PZT sensor is placed, with the real component of the impedance often used in the analysis [25,30,32,34,38]. The relationship between the electrical and the mechanical variables for a linear piezoelectric material can be described by linear relations [41,76]. The impedance method measures the effective resistance of the structure through the use of high-frequency structural excitations of the structure-bonded transducer patches to detect any changes in the host structure’s mechanical impedance [25,77,78]. Damage is evaluated by comparing measurements from active transmission at a time in its lifespan to the baseline data of the undamaged structure to determine the damage to the structure. The detection is possible because the damage close to the sensor causes a variation in stiffness and affects the structure’s resonant parameters, which will, in turn, change the electrical impedance of the transducer accordingly, owing to the electromechanical coupling [22,36,38,75]. For high sensitivity to damage, the electrical impedance is measured at high frequencies (e.g., 30–400 kHz) to ensure that the wavelength of the excitation is small and sensitive to minor structural integrity [22,31,42,79].

### 3.2. Applications of the Piezoelectric Impedance Sensors in SHM

The impedance-based method has been experimentally implemented in various case studies ranging from one dimensional to two-dimensional cases related to trusses, beams, concrete, composites, etc. [25,38,39,41,43,45,77,80,81]. The performance of the piezoelectric impedance-based method has been validated by laboratory experiments such as damage detection for composite-reinforced concrete walls and the detection of a loosening bolt on an experimental three-story moment-resisting frame structure, adhesive defect monitoring of lass fiber epoxy [38,44]. Figure 2b displays how the PZT sensors are installed to monitor a structure via measuring impedance variations. Figure 2c presents the variations in the measured impedance outputs for SHM. A practical approach to detect damage in this method is to simplify the data using a damage metric chart, as shown in Figure 2d [46,81]. The damage metric chart is plotted to assess the structural health status through comparison with the baseline data [35,36,46]. As seen in Figure 2c, the crack frequency changes from the baseline in loading 1 and 2. This change in impedance indicates the occurrence of damage, and it is usually expressed using the root mean square deviation (RMSD) statistical algorithm given in Equation (5) [31]

(5) M=∑i=1n[Re(Zi,1)−Re(Zi,2)]2[Re(Zi,1)]2

where M represents the damage metric; *Z_i_*_,1_ is the impedance of the PZT measured at healthy conditions; and *Z_i_*_,2_ is the impedance for the comparison with the baseline measurement at frequency interval *i*. Other scalar damage metrics include mean absolute percentage deviation (MAPD), covariance change (CC), correlation coefficient deviation (1−R^2^), and third power of the correlation co-efficient derivative have also been utilized in SHM applications [30,33,35,80,82].

It should be noted that the sensors are prone to damage, which may result in faulty detection. As a result, some researchers have developed sensor diagnostic processes that can be efficiently used to ascertain the working condition of the sensing network [42,83,84]. As an example, Figure 2e shows an experimental study on a pipeline consisting of three pipe sections connected using flanged joints to form a continuous pipeline [40]. Damage is induced, and impedance measurements are used to detect damage occurring at pipeline joints. Flexible macro-fiber composite (MFC) patches can be used as both sensors and actuators to record electrical impedance signal before and after the damage [24,53]. The location of joint damage can be successfully determined from the measured responses. The cross-correlation coefficient damage metric is used to compare data between a particular damage case and the baseline measurement to ascertain the degree of damage [32,46,54].

## 4. Piezoelectric Guided Wave Ultrasonic Technique

Guided waves are an essential category of mechanical waves. Guided waves are of particular importance in SHM because of their low energy loss, long propagation range and ability to travel inside curved walls. These features make them well suited for the ultrasonic inspection of tubular structures such as aircraft, pressure vessels, missiles, and pipelines. Guided waves include Rayleigh wave, a particular case of shear-horizontal (SH) waves and Lamb waves [55]. The Rayleigh waves travel close to the free surface with very little penetration in the solid depth. As a result, Rayleigh waves are also known as surface-guided waves. SH waves have particle motion contained in the horizontal plane defined between the *x* and *z*-axis. The focus of this study is on Lamb waves. These waves are guided between two parallel free surfaces, such as the upper and lower surfaces of plates and shells. Lamb waves exist in two basic modes symmetric and antisymmetric. They are highly dispersive and their speed depends on the product of frequency (*f*) and the plate thickness (*t*), i.e., (*f × t*). As *f × t* approaches infinity, the Lamb wave modes change into Rayleigh waves [55].

### 4.1. Design Principle and Paradigms

**Figure 3:**
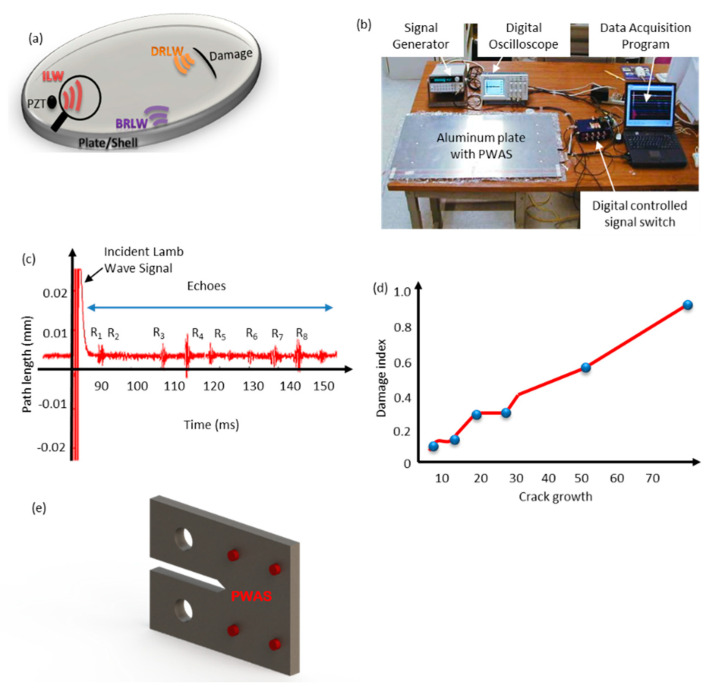
(**a**) A cracked aluminum plate showing incident Lamb waves (ILW), boundary reflected Lamb waves (BRLW) and damage reflected Lamb waves (DRLW) [51,52], (**b**) Experimental setup showing the plate, active sensors, and instrumentation [51,52], (**c**) the incident signal and the reflected (echoes) signals on sensor [51,52], (**d**) Damage Metric [58] (**e**) A CT specimen with PWAS.

Horace Lamb, in 1882, proposed the propagation of two sets of wave modes propagating in an infinite medium. The so-called Lamb wave can be induced in plates and shells at specific excitation frequencies, as illustrated in Figure 3a. The Lamb wave properties are complex, and its velocity depends on the thickness of the plate [55,85]. In reality, no plate is infinite, and Lamb waves are bounded by the plate or shell in which they propagate, leading to a waveguide effect. Hence, this type of wave is generally called a “guided” lamb wave [55,85]. While the lamb wave method is popular in the area of non-destructive evaluation, it has some limitations. Some of the challenges are the difficulties in generating specific wave modes with high enough amplitude, achieving a proper propagation, and clear “echoes” in return from defects. Therefore, a high degree of skill and careful excitation is required to deploy this method. A second challenge is that mode conversion can take place as a result of reflection from the defects. This may result in difficulty in the interpretation of results [47]. Lamb waves can be induced in materials in several ways, such as electromagnetic acoustic transducers (EMAT’s) and comb transducers. In this paper, we focus on the studies that utilize piezoelectric transducers for inducing Lamb waves. We present the most widely used approaches based on piezoelectric wafer active sensors (PWASs) and Stanford Multiactuator–Receiver Transduction Technology (SMART).

### 4.2. Applications of the Ultrasonic Lamb Wave Technique in SHM

#### 4.2.1. PWAS Technology

The PWAS technology uses piezoelectric material to generate Lamb waves and detects the reflected Lamb wave using the pulse-echo method. Lamb waves propagate in solid plates, and the reflected Lamb wave can be analyzed to detect damages, as shown in Figure 3b,c [43,47,53,55,85]. In the area of SHM, research work on Lamb wave has been majorly focused on two aspects: Lamb wave excitation and detection [86,87,88,89,90,91,92], and its application in damage detection [48,49,93,94,95,96,97,98,99,100]. Researchers have applied this method to a wide range of problems such as corrosion detection, bolt loosening, crack detection, and delamination [101,102,103,104,105,106]. More innovative approaches have focused on developing computational models to simulate piezoelectric-actuator-induced acoustic-ultrasonic wave propagation [50,107,108]. The principle of Lamb wave generation and detection by PWAS probes have been verified in laboratory experiments for the detection of cracks [51,52]. The principal modes of PWAS-induced Lamb waves have been extensively studied by researchers [47,56,100,109,110,111,112,113]. This method has been used as a multi-mode sensing system for corrosion detection, delamination in composite laminates, and the identification of damage in plate-like structures [57,58,59,60]. Lamb waves can be tuned using varying symmetric and antisymmetric excitation frequencies to enable the PWAS to detect specific kinds of defects in structures. Symmetric Lamb waves receive a stronger echo from a through-the-thickness crack than antisymmetric waves. The antisymmetric Lamb wave mode is more sensitive to debonding, delamination, and corrosion [51,61]. The PWAS working mechanism is simple. The sensor is electrically activated after being bonded to the host structure. A strain is then induced in the PWAS as a result of the electrical charge. The interaction forces at the interface between the sensor and the structure create Lamb waves [47,51,100].

When an elastic wave propagates through the structure, the sensor is activated through the strain/displacement compatibility condition. The strain induced in the sensor generates an electric field that is captured as voltage at the sensor terminals [46,47,92,93,94,95,96,97,98,99,100,101,102,103,104]. Consider an array of three active sensors acting as both actuators and receivers, as one sensor acts as an actuator (“A”), the others act as sensors (“B” and “C”). PWAS “A” generates elastic waves propagating through the structure and detected by sensor “B” and “C.” The sensed waves’ characteristics are affected by the presence of damage and other structural features, which are then analyzed to detect the damage location. The system is rotated to sense other areas with sensor “B” and “C,” taking turns to generate waves with the rest of the sensors being wave receivers [52]. As a rule of thumb, Lamb waves are guided by the boundaries of the solid materials media, which they propagate in [86,87,88,89,90,91,92]. The pulse-echo method and pitch-catch method are the basis of several non-destructive guided wave testing. This sensor uses these methods to creates electrical pulses that are later transformed into mechanical vibrations by the PWAS, as shown in Figure 3b [55]. Based on the reflection echoes and sound velocity in the material, discontinuities in the sample can be detected.

Figure 3c shows a typical result of the mechanism of lamb wave excitation and detection. The initial pulse and Reflections (echoes R1 to R8) can be seen in this figure. As an example, Figure 3d shows a thick steel structure where PWAS has been used to perform an active and non-destructive evaluation of a fatigue crack using the guided wave pitch-catch method. The study included a finite element analysis and experimentation that can be found in [58]. The experimental procedure included fatigue testing of compact tension (CT) specimens that are commonly used in fracture mechanics. In such experiments, PWAS is bonded to the specimen to excite and receive the guided wave and to detect and quantify the crack length. The damage index is then quantified against crack growth, as shown in Figure 3e.

#### 4.2.2. SMART Technology

**Figure 4:**
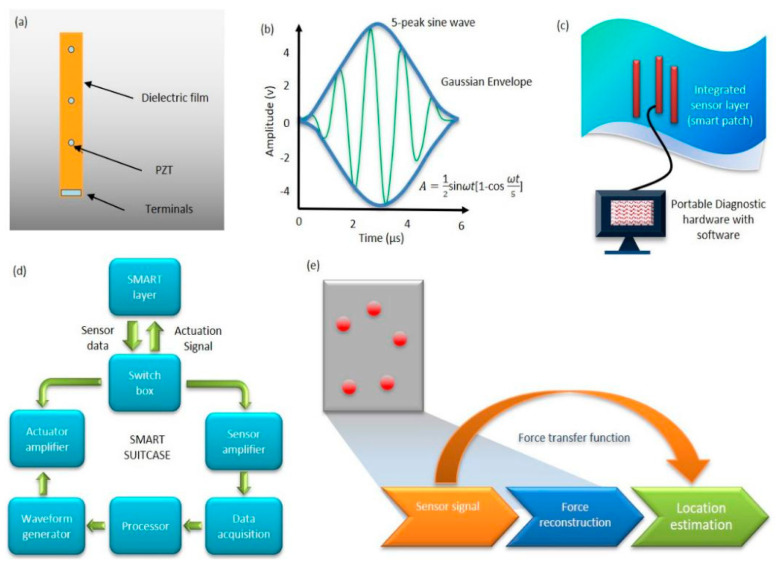
(**a**) The Stanford Multiactuator Receiver Transduction (SMART) Layer Technology [64], (**b**) 5-peak sine Waveform [63], (**c**) Thermal protective system (TPS) panel with smart sensor [62], (**d**) Smart Suitcase workflow [63], and (**e**) Overview of impact identification scheme [68] (Courtesy of Acellent).

The SMART technology is patented to Acellent Technologies, Inc., Sunnyvale, CA, USA. It is composed of the SMART layer shown in Figure 4a and the SMART suitcase^TM^. The technology has been tested for its effect on structural integrity [62]. The feasibility of fabricating SMART layers into 3D shapes, their surface mounting, and implantation within composite and integration into a filament-wound structure has been investigated [63,64]. Several studies have been conducted to evaluate the effects of loading, temperature, moisture, and aggressive chemical environments on SMART technology [62,63,64,65,66,67,68]. The SMART layer is fabricated based on the flexible printed circuit technique. The layer consists of a thin dielectric film embedded with an array of piezoelectric actuators/sensors intended to be surface-mounted on metallic structures or embedded inside composite structures. Figure 4c is a mobile diagnostic unit developed to interface with the SMART layer to generate interrogative signals from actuators and detect measurements from the sensors [65]. It can also carry out passive monitoring by sensing waves from impact. A method for identifying impact events using a built-in interrogative technique can be utilized with the piezoelectric sensors (Figure 4d). The method includes impact location and force-time history.

The SMART layer technology has been deployed in many case studies because of its remarkable versatility in composite and metallic materials [62]. For instance, the layer can be placed in between the composite and foam core at the manufacturing phase of the composite foam core structure to monitor it in-service [62,64]. In aerospace applications, this method has been used as an on- and off-board SHM technology [62,69]. It has been successfully applied to the detection of impact damage in a composite pressure vessel, large composite fuselage barrel, full-scale crewless combat air vehicle (CCAV) composite wing, and composite rocket motors, as well as monitoring crack growth under bonded repair and in riveted lap joints in metallic structures [62]. Figure 4e illustrates a typical SMART layer impact identification scheme for the passive diagnostic of large composite structures under impact loading. An external impact created by a hammer is applied to a specimen of a thermal protection system (TPS) panel. The generated mechanical stress waves propagate on the surface of the TPS panels to reach the PZT sensors. The location and impact force can be determined by analyzing the sensor response [62,70].

## 5. Piezoelectric-Floating-Gate Sensing Technology

### 5.1. Design Principles and Paradigms

**Figure 5:**
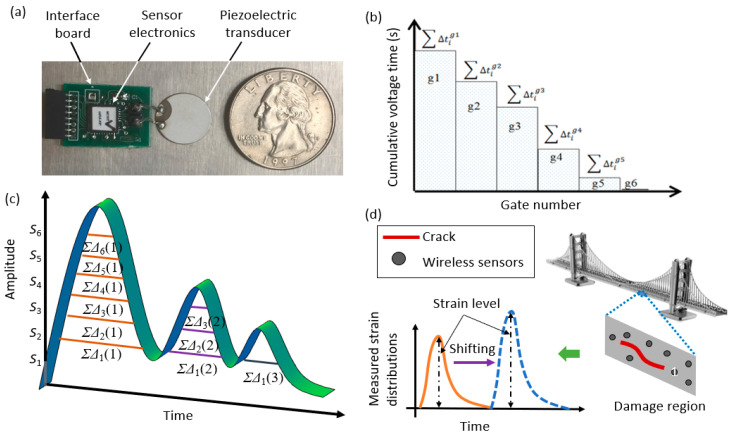
(**a**) Components of a piezoelectric-floating-gate (PFG) sensor, (**b**,**c**) Multi-level counting of cumulative times based on the strain values, and (**d**) Shift in probability density functions (PDFs) as damage progresses in a bridge [72].

The piezoelectric-floating-gate (PFG) technique developed by the teams at Michigan State University and Washington University in St. Louis comprises a floating-gate unit interfaced with a piezoelectric transducer [71,114,115,116,117]. The PFG sensors have achieved the power consumption of at least two orders of magnitude lower than that of other sensing technologies (i.e., 80 nW power consumption for the latest prototype). Figure 5a demonstrates the prototype of the PFG sensor [115]. The electronics that make up the floating gates are approximately 1.5×1.5 mm, packaged into a standard 6×6×0.8 mm quad-flat no-leads (QFN). The piezoelectric transducer next to the PFG sensor is used to harvest electrical energy from the host structures’ mechanical response. The following equation can be used to determine the piezoelectric generated voltage V [71]

(6) $$V=\frac{SEd_{31}t}{\varepsilon }$$

where:

*S*: Strain;
*E:* Piezoelectric material Young’s modulus;
*d*_31_: Piezoelectric constant;
*t:* Piezoelectric thickness;
ε: Piezoelectric electrical permittivity.

The PFG sensor consists of a series of memory gates that are next to the computational circuits. The detected information obtained by the piezoelectric transducer is transferred and stored in those gates. The duration of the transducer’s voltages (i.e., electrical signals) are recorded in the gates when the amplitude exceeds predefined thresholds, and the stored cumulative durations are regularly read using Radio Frequency Identification (RFID) scanners. The outputs of the PFG sensors are obtained in terms of histograms, which use the bins as a cumulative time for the events at specific strain levels [117].

The PFG sensors possess various advantages because of their “response-based” characteristics. The entire methodology is based on relative damages, since the sensors do not directly measure the absolute values of strains. Instead, the variation rates of strain distributions are related to the damage rates. Figure 5b displays the strain history measured by the PFG sensors, in which each gate number corresponds to a predefined voltage/strain level. Figure 5c illustrates the charge generated by the transducer due to the mechanical response of structures. According to the previous studies [15,71,72,73,114,115,116,117], the sensor outputs are characterized by a cumulative density function (CDF) or probability density function (PDF) as [113]

(7) $$\mathrm{CDF}\left(g\right)=\frac{\alpha }{2}\left[1-\mathrm{erf}\left(\frac{g-\mu }{\sigma \sqrt{2}}\right)\right]$$

(8) $$\mathrm{PDF}\left(g\right)=\frac{1}{\sigma \;\sqrt{2\;\pi }}\;e^{-\frac{{\left(g-\mu \right)}^{2}}{2\;\sigma^{2}}}$$

where, *µ* and *σ*, respectively, denote the mean of cumulative time distribution of the applied strain and standard deviation accounting for the load and frequency variability. *α* and *g*, respectively, represent the total cumulative time of the applied strain measured by all floating gate units and the number of floating-gate units each corresponding to a pre-defined strain level. Structural damage progressions can be accurately captured using the *µ* and *σ* parameters [117]. Figure 5d schematically illustrates the application of the PFG sensors in the detection of damage on a bridge gusset plate. Since the PDF sensors are distributed over targeted regions in the structure, the structural conditions can be detected using the relative variations in the strain response in the PDF. In other words, structural damages are detected by tracking the shifts in the PDF outputs (i.e., *µ* and *σ*) over time, rather than directly measuring structural damages [72].

### 5.2. Applications of PFG in SHM

**Figure 6:**
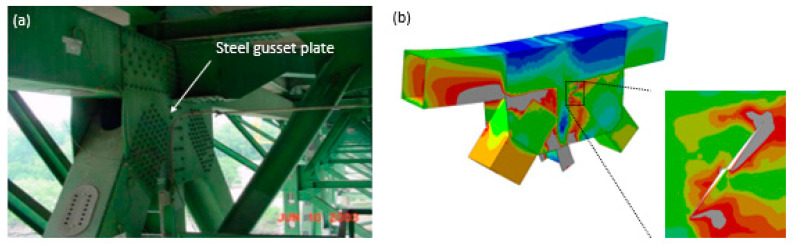
(**a**) A gusset plate structure, (**b**) finite element (FE) model of the failed gusset plate [15,73,74].

The PFG sensors aim to provide a cost-effective continuous, and reliable assessment of the structural condition. This technology has been used for many SHM applications such as failure of concrete beams, crack growth detection in steel plate, distortion-induced fatigue cracking in bridge girders, failure of gusset plates, and bolt loosening in structures with bolted connections [15,71,72,73,114,115,116,117]. As an example, Figure 6a presents the U10W gusset plate, which was a part of the collapsed I-35W Highway Bridge in Minnesota. Previous studies have applied the PFG sensors to detect crack growth in this gusset plate [15]. The gusset plate finite element (FE) model is shown in Figure 6b. The PFG sensing mechanism has been used to predict the damage progression on such a gusset plate via extracting the strains at hundreds of nodes. The CDF was used to fit the cumulative data, which were then transformed into PDFs. However, the distribution patterns of the PDF curves were different between sensors, and therefore, the effective sensor fusion model was developed to enhance the damage progression identification through spatial measurement. The reported sensor fusion process integrated and extracted useful information from multiple sensors. It has been reported that the fused features obtained from the PDFs are proportional to the damage status [15,73,74].

## 6. Methods of Power Delivery to SHM Sensor Nodes

In an SHM system, one of the critical challenges of continuous monitoring is securing a sustainable power resource. Sensor nodes can be powered via cables connected to a power source, built-in batteries, wireless power transfer (WPT), and energy harvesting (EH). The cabled and battery-powered approaches are the most commonly uses methods in SHM. In cabled networks, each sensor is connected with a wire to power the sensor, which makes the sensing area clustered. Battery-powered sensors use small dry cells to power each sensor. They are mostly implemented in significantly small sensors (coin-sized) and can wirelessly transmit data using a low-power radio signals [14,118]. Most research in electromechanical impedance and guided ultrasonic lamb waves sensing employs either a wired or a battery-powered approach. Arguably, there is a need to enhance this SHM methods with WPT or EH capabilities. The WPT or EH methods can readily empower more sensor nodes without the need to change batteries regularly or have a wire-clustered sensing area. The WPT methods can be categorized into radio frequency (RF) transfer, optical power (OP) transfer, ultrasonic transfer, capacitive power (CP) transfer, and inductive power (IP) transfer [119]. The RF transfer entails the use of radio signals in the frequency range of 300 GHz to 3 kHz to carry energy wirelessly over a reasonably long range with the aid of a transmitter and receiver [120,121]. The OP transfer makes use of light in the ultraviolet, visible, and infrared bands of the electromagnetic spectrum to transfer energy from the source to receiver over long distances. Sources include power laser diodes and sun, while receivers include solar cells, photovoltaic converter, or photodiode [119]. The ultrasonic power transfer systems consist of ultrasonic transmitters and the receiving nodes being powered. Most ultrasonic power transfer systems bear a resemblance to vibration-based energy-harvesting systems. They use piezoelectric materials for the conversion of ultrasonic signals from a transmitter into electrical energy in a sensor node in a short transfer distance [122,123,124]. The CP transfer method utilizes a time-changing electric field between capacitor terminals to transfer power via air. On one end of the capacitor, power is converted into an excitation form to excite a transmitter device. It generates a corresponding energy field and transmits it to the second terminal to power a load. This method is mostly suited to low power levels and across small distances in magnitudes of mm [119,125,126]. The IP transfer occurs as a result of mutual magnetic coupling. A change in the current through at one terminal induces a voltage across the other terminal’s ends through electromagnetic induction. Power transfer occurs via a mutual flux linking primary and secondary coils, enabling power transfer to a resistive load, creating a voltage [119,124,126]. Considering the ease of implementation, the most suitable WPT method for SHM methods are the RF transfer (long-range WPT) and ultrasonic power transfer (short-range WPT) approaches.

While it is feasible to harvest the mechanical energy from the ambient vibration using piezoelectric materials, the scavenged energy is on the order of microwatts to milliwatts and is merely suitable for empowering low-power electronics. When compared to thermal and solar energy harvesters, which can generate hundreds of watts, piezoelectric materials usually operate at much lower energy levels. Accordingly, the piezoelectric elements included in the reviewed electromechanical impedance and ultrasonic Lamb wave SHM methods mainly serve as the sensing medium and not a sensor-empowering resource. It should be noted that the piezoelectric transducers in the PFG sensors could have also been used as power energy harvesters because of the ultra-low power consumption of the interfaced floating-gate computational circuits [71,114,115,116,117].

## 7. Discussion: Piezoelectric Sensing Techniques for Smart and Connected Civil Infrastructure

Table 2 summarizes the pros and cons of the piezoelectric electromechanical impedance, ultrasonic Lamb wave and PFG methods.

**Figure 7:**
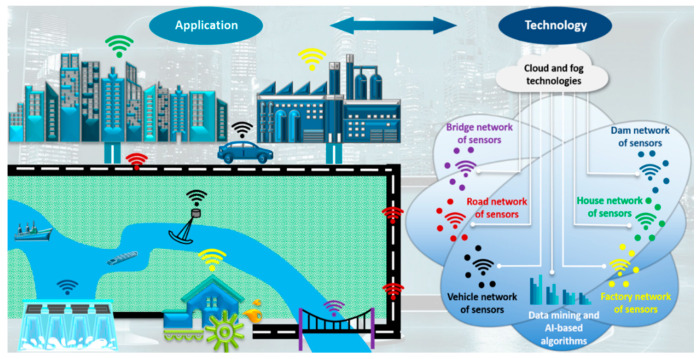
A vision for next-generation smart and connected civil infrastructure platform integrating advanced SHM, information, and data mining technologies.

However, modern civil infrastructure systems are designed to achieve advanced functionalities for multipurpose applications under critical conditions such as earthquakes, storms and typhoons [127]. There are serious concerns about the integrity of such complex structural systems. A viable solution to this issue is to integrate a network of smart and embeddable sensors into civil infrastructure systems with local artificial intelligence (AI)/machine learning (ML) data-processing frameworks, enabling next-generation smart, civil infrastructure systems built on the traditional systems’ skeleton. The features of the piezoelectric sensing techniques reviewed in this study make them an ideal option. A substantial portion of the research in this area has gone into the SHM of bridges and pavements. Arguably, more research is needed to harness the capabilities of the piezoelectric sensing methods in other critical structural systems such as flood defenses and dams. Figure 7 envisions a smart, civil infrastructure platform with an integrated piezoelectric-based SHM functionality. In this platform, structures can wirelessly communicate health status, allowing for prompt assessment of the status and safety of those structures. However, a significant challenge would be to process the massive amount of information generated by the sensor networks, necessitating an enhanced smart, civil infrastructure platform with Big Data and cloud and edge/fog computing and internet-of-things (IoT) capabilities. Incorporating all of these features will create an ingenious and connected system that allows enhanced data-sharing and decision-foreseeing for the civil infrastructure systems. Besides, piezoelectric sensing techniques can play an essential role in maintaining the functionality of civil infrastructures under extreme conditions over a relatively long time. This topic needs remarkably more research. However, compared to other SHM sensing systems, piezoelectric sensors are still reliable and can be applied in different environments upon proper calibrations.

With the development of EH techniques, wireless piezoelectric sensors have been expanded to self-powered sensing systems with high stability and sustainability in different SHM applications. Future smart civil infrastructures are expected to be capable of self-monitoring and self-charging by integrating the piezoelectric nanogenerators and ultra-low power consumption circuits. Achieving these goals requires improving the piezoelectric sensing systems in the following aspects:

▪ The ability to perform spatial SHM over large structural areas without a need to deploy hundreds of sensing nodes;
▪ Enhancing sensitivity to various damage types;
▪ On-board computation algorithms;
▪ Reliable wireless communication techniques;
▪ Enhancing the power density of piezoelectric sensors for self-powering applications;
▪ Integrating the sensor with different plug-in functionalities;
▪ Reducing the deployment cost via material design, optimal sensor placement strategies, etc.

## 8. Conclusions

This study presented an overview of piezoelectric sensing techniques for SHM systems. We reviewed techniques ranging from piezoelectric impedance and ultrasonic Lamb wave-sensing to self-powered systems. The principle of each method and its applications in assessing structural health condition were studied. The methods are evaluated based on their sensing and design functionalities. Further discussion about the future trends of piezoelectric sensing implied that this sensing technology can play an important role in the SHM systems of next-generation smart and connected civil infrastructure platforms. More research is needed to harness the capabilities of piezoelectric sensing techniques for other large-scale infrastructure systems. This will mainly involve finding solutions for spatial monitoring with less sensing nodes and enhancing the energy harvesting efficiency for more advanced self-powering applications, particularly for embedded systems.

## Figures and Tables

**Table 1:** An overview of studies focused on using the piezoelectric sensing techniques in SHM.

Sensing Mode	Sensor Type	Principle	Existing Studies
Electromechanical impedance	Piezoelectric Impedance Transducers	Measuring effective resistance of structures and comparing to baseline data	[18,19], [24,25], [30,31,32,33,34,35,36,37,38,39,40,41,42,43,44]
Guided Lamb Wave	Piezoelectric wafer active sensor	Generating Lamb waves and detecting reflected Lamb wave using pulse-echo method	[45,46,47,48,49,50,51,52,53,54,55,56,57,58,59,60,61]
	Stanford multi-actuator–receiver transduction technology layer	Generating ultrasonic signals from actuators and detect measurements from sensors	[62,63,64,65,66,67,68,69,70]
Electrical signals	Piezo-floating-gate	Recording piezoelectric transducer electrical signals above predefined thresholds	[15,71,72,73,74]

**Table 2:** Pros and cons of the piezoelectric electromechanical impedance, ultrasonic Lamb wave and PFG methods for SHM applications.

Sensor Type	Suitability	Pros	Cons
Piezoelectric-Impedance Technique	• Suitable for a broad spectrum of civil infrastructure systems; • Applicable to concrete, metallic and other fiber composite structures.	• High sensitivity to inchoate damages as a result of the high-frequency detection mechanism; • Measures a broad spectrum of defects such as crack growth, deboning, corrosion, loosening bolts; • Data interpretation is relatively easy; • It is not very sensitive to structural boundaries.	• Wired; • Due to high-frequency detection, an effective range of detection is reduced; • The use of a network of sensors to adequately monitor a given area is required. • An external power source is required, e.g., a battery.
Piezoelectric Guided Wave Ultrasonic Technique	• Most suitable for metallic plates and shells. It can also be applied to thin fiber composites.	• Lamb wave modes vary in their sensitivity to damages, i.e., a given mode may be more sensitive to specific damage than others; • Lamb wave modes can be tuned at certain frequencies, which permits specific modes to be generated for the primary purpose of detecting a particular type of defect; • Can be used to monitor a large area.	• Wired; • Very sensitive to frequency and can be complicated to excite and interpret its data especially when a single mode is required for damage interrogation; • Mode conversion may occur as a result of lamb wave reflection from damage; • External power is required, e.g., a battery; • Very Sensitive to reflection from boundaries, which should be taken into account during damage detection.
PFG Technique	• Most suitable for steel and metallic structures but may also be used for concrete structures.	• Wireless; • Self-Powered; • Inexpensive; • Easy installation.	• Data interpretation is relatively complicated.
